# The potential application of platelet-rich fibrin (PRF) in vestibuloplasty

**DOI:** 10.1186/s40902-021-00308-4

**Published:** 2021-07-01

**Authors:** Mohammad Amin Amiri, Nima Farshidfar, Shahram Hamedani

**Affiliations:** 1 Student Research Committee, Shiraz University of Medical Sciences, Shiraz, Iran; 2 Oral and Dental Disease Research Center, School of Dentistry, Shiraz University of Medical Sciences, Shiraz, Iran

**Keywords:** Platelet-rich fibrin, PRF, Membrane, Vestibuloplasty, Oral surgery

To the Editor,

Since removable dentures are a common treatment for optimal improvement in function and esthetics in patients with edentulism [1], pre-prosthetic surgeries—especially vestibuloplasty procedures—play an imperative role in preparing the oral environment for a better denture stability [2]. Vestibuloplasty surgeries are usually performed to provide adequate depth in labial or lingual vestibular area for denture flange [2]. Regarding the procedural aspect, several techniques have been proposed such as Kazanjian technique [3] and lip-switch technique [3]. All these techniques entail the presence of excessive mucosa to enhance the vestibular depth. In Kazanjian technique, this issue is addressed by providing the excessive mucosa from labial mucosa which consequently leads to exposure of labial side of the vestibule [3]. To compensate for this complication, in lip-switch technique, incision and dissection of periosteum is performed, and the periosteum is placed over the denuded labial submucosa [3]. However, this modified technique also requires excessive incisions to provide more mucosa. Moreover, in order to cover the exposed area in other vestibuloplasty procedures, several autogenous grafts such as skin or mucosal grafts have been employed, although each one has its own advantages and disadvantages [2].

In 2000, platelet-rich fibrin (PRF) as the second generation of autologous platelet concentrates (APCs) caught extreme attention for various oral and maxillofacial surgery applications [4]. This natural biomaterial can be easily prepared by drawing a 9–10 ml blood sample and then performing centrifugation [5]. Despite the other generations of APCs, PRF has no anti-coagulant agents and has exerted excellent anti-bacterial [5], anti-inflammatory [5], and regenerative potentials [5]. Regarding the unique characteristics of this blood-derived product, it has been broadly used in various oral and maxillofacial surgery procedures such as socket preservation [6], ridge augmentation [5], dental implant placement [5], and so on [5]. In this regard, PRF has been applied to enhance epithelialization to avoid mesh exposure which has been reported as a complication in some surgical procedures such as ridge augmentation [7] and cranioplasty [8]. Furthermore, PRF has been utilized to enhance epithelial keratinization and to heal the wound in palatal donor site after harvesting the free gingival grafts (FGG) [9]. These regenerative characteristics of PRF have led to favorable outcomes in regeneration of epithelium in the reported cases [7, 9]. This is mainly ascribed to the ability of the fibrin network to release different types of growth factors which can also serve as an ideal medium for adhesion, migration, proliferation, and differentiation of various cells [5].

To reduce the shortcomings of conventional vestibuloplasty procedures, PRF membrane can be employed for covering the exposed area. Therefore, there would be no need for further skin or mucosal graft harvesting; besides, the epithelialization rate of the wound can be accelerated [5]. The ease-of-applicability of this natural biomaterial can ultimately increase its acceptance among surgeons and patients, while it leaves patients with less complications after surgery. However, since PRF is a fragile and mechanically weak biomaterial, its fixation to adjacent tissues is a frequent issue and it may be susceptible to be torn off during the suturing process. In this regard, in order to make a proper adaptation in the grafted area without any additional tension in the PRF membrane, the following approaches can be proposed and implemented: application of various tissue bioadhesives (e.g., cyanoacrylate) [10], placement of stents over the grafted area [11], or employment of different suturing techniques (e.g., crisscross suture) [12] which can only hold the PRF membrane in the grafted site without any increased tension in the PRF. Therefore, regarding the above-mentioned facts, we propose that PRF could be an acceptable alternative to autogenous grafting in vestibuloplasty procedures. Nevertheless, many strong clinical trials should be performed to demonstrate whether this intervention would improve the surgical outcomes and satisfy the involved clinicians and surgeon in the real clinical situations.

## Data Availability

Not applicable

## Abbreviations

APCs: Autologous platelet concentrates
FGG: Free gingival grafts
PRF: Platelet-rich fibrin
